# Percolation Characteristics and Injection Limit of Surfactant Huff-n-Puff in a Tight Reservoir

**DOI:** 10.1021/acsomega.2c03679

**Published:** 2022-08-15

**Authors:** Guangsheng Cao, Qingchao Cheng, Hongwei Wang, Ruixuan Bu, Ning Zhang, Qiang Wang

**Affiliations:** † Key Laboratory of Enhanced Oil & Gas Recovery of Ministry of Education, Northeast Petroleum University, Daqing 163318, P. R. China; ‡ Daqing International Exploration and Development Company, Daqing 163000, China; § No. 2 Oil Production Plant, Daqing Oilfield Co., Ltd., Daqing 163000, China; ∥ Research Institute of Exploration and Development of Daqing Oilfield Company Ltd., Daqing 163000, China

## Abstract

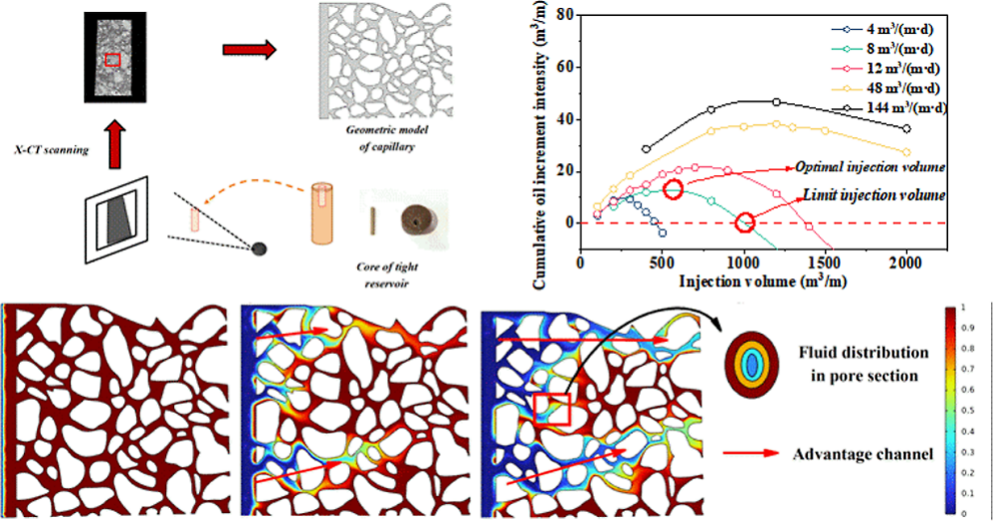

For the development of tight reservoirs, large-scale volume fracturing is frequently utilized as an effective production enhancement strategy. However, there is a significant decrease in productivity after fracturing. Improvement of production through secondary surfactant huff-n-puff has become one of the methods. In this paper, the characteristics of surfactant percolation during huff-n-puff were analyzed from macroscopic and microscopic perspectives. The production variation characteristics of the huff-n-puff were calculated by experiments and numerical methods. From Stokes’ equations and phase field equations, solutions were found to analyze the effect of interfacial properties on surfactant percolation from the microscopic perspective. The findings demonstrated that a surfactant with a high displacement efficiency could not considerably increase huff-n-puff production, whereas the percolation rate had a wider influence. The surfactant with ultralow interfacial tension (<1 × 10^–2^ mN/m) and a higher wetting angle (>12.6°) has a faster percolation rate. Significant huff-n-puff production can be obtained in the percolation rate range of 1.38 to 1.63 m/PV. Simultaneously, the concepts of limit and optimal injection volume were established and utilized to characterize the influence of injection parameters on production under nonextension fracture situations. Based on the data, in order to obtain high production in a short time, the injection strength should be near to the value at fracture extension, and the optimum injection volume is 1000–1200 m^3^/m. The findings of this study have the potential to guide the selection of the surfactant and injection parameters in the field.

## 1 Introduction

With the continuous decline of the conventional reserves, exploration of the tight and low permeability reservoirs has become a trend.^1,2^ Hydraulic fracturing stimulation has emerged as a critical method for extracting these types of hydrocarbon resources.^3,4^ The purpose of hydraulic fracturing is to increase the fluid seepage area and convert radial flow into linear flow in order to reduce seepage resistance. The surfactant is also widely utilized in oil production of low-permeability reservoirs as a low-cost working fluid that can reduce oil–water interfacial tension, change wettability, and increase oil–water emulsification.^5−10^ Several studies indicate that fracturing fluid imbibition on the reservoir fracture surface is one of the core processes of enhanced oil recovery (EOR).^11−16^ Through the visualization model experiment, some researchers have shown that when the interfacial tension is reduced to ultralow, the surfactant and oil generate microemulsions during the displacement process.^17,18^ The imbibition action of the microemulsion can force the oil into the tight matrix.^19^ According to Sharma and Sheng et al.,^20,21^ the imbibition of a low permeability core in surfactant solution (IFT is around 1 mN/m) is a reverse flow process by CT scanning (oil flows out of the pores), implying that capillary force dominates the process. Tagavifar et al.^22^ used numerical simulation to reveal surfactant adsorption kinetics. They noticed that emulsification at the appropriate salinity promotes early wettability changes. Adding the surfactant to the fracturing fluid can reduce the capillary retention of the fracturing fluid in tight reservoirs.^23^ These theoretical studies convincingly demonstrate how surfactants enhance oil recovery during the fracturing process. Similarly, surfactant huff-n-puff is frequently required due to the inadequate fluid percolation capacity in tight reservoirs and the short production time after fracturing. The percolation area of the surfactant in the huff-n-puff process is commonly more essential than just reducing interfacial tension.^24^ Chabert et al.^25^ demonstrated that the dynamic phenomenon between the cracks and matrix plays a significant role in surfactant EOR efficiency. Abbasi-Asl et al.^26^ observed that although surfactants with low interfacial tension lack the promotion of capillary forces, the transverse pressure gradient promotes surfactant transmission to the matrix at a feasible rate. Kamath et al.^27^ indicated that low-pressure flooding could reduce interfacial tension.

Scholars mostly debate the stimulation mechanism of surfactant fracturing. Little research has been conducted to investigate the association between surfactant percolation capacity and huff-and-puff productivity. The macroscopic and microscopic characteristics of surfactant percolation were studied in this paper. The production variation was calculated using huff-and-puff experiments and numerical simulations. By solving the Stokes’ equations and the phase field equations, the effects of interfacial tension and wetting angle on surfactant percolation were investigated from a microscopic standpoint. Similarly, the experimental scale surfactant percolation capacity and the injection limit under actual production conditions are quantified. The limiting and optimal injection volumes, as well as the optimal surfactant percolation rate range, were provided. The findings of this paper have significant implications for the development of tight reservoirs.

## 2 Methodology

### 2.1 Fluid Flow Equation in Porous Medium

**Figure 1:**
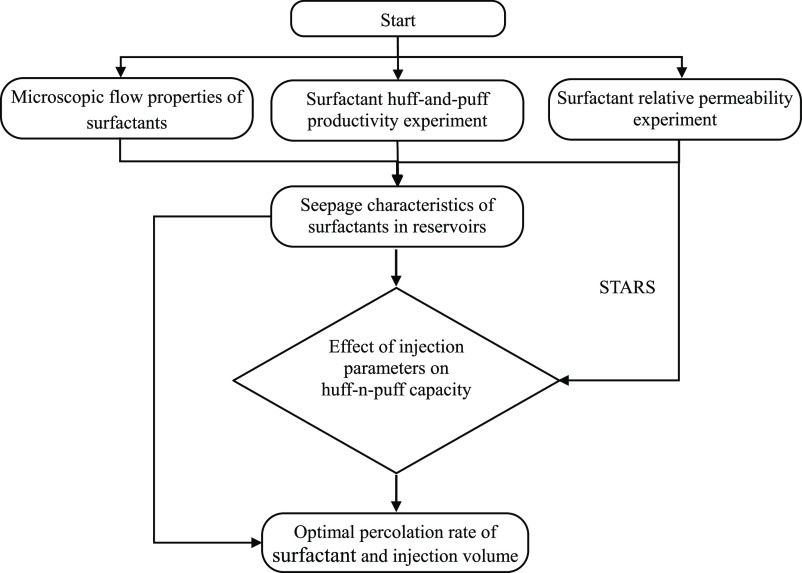
Working schematic of the research.

The investigation was performed from a microscopic perspective to demonstrate the percolation features of surfactants during huff-and-puff. Figure 1 depicts the study’s schematics. When surfactants are injected into the reservoir process, changes in the interfacial characteristics of the surfactants alter the percolation zone of the fracturing fluid. If the surfactant percolates far from the well, the fluid cannot be effectively propelled by the production pressure differential, decreasing the surfactant’s ability to convey the oil. Furthermore, if the percolation is too close, the well drainage area cannot be extended by the extra energy. As a result, it is required to calculate the microscopic flow characteristics of surfactants in porous media and identify the surfactant parameters appropriate to tight reservoirs.

**Figure 2:**
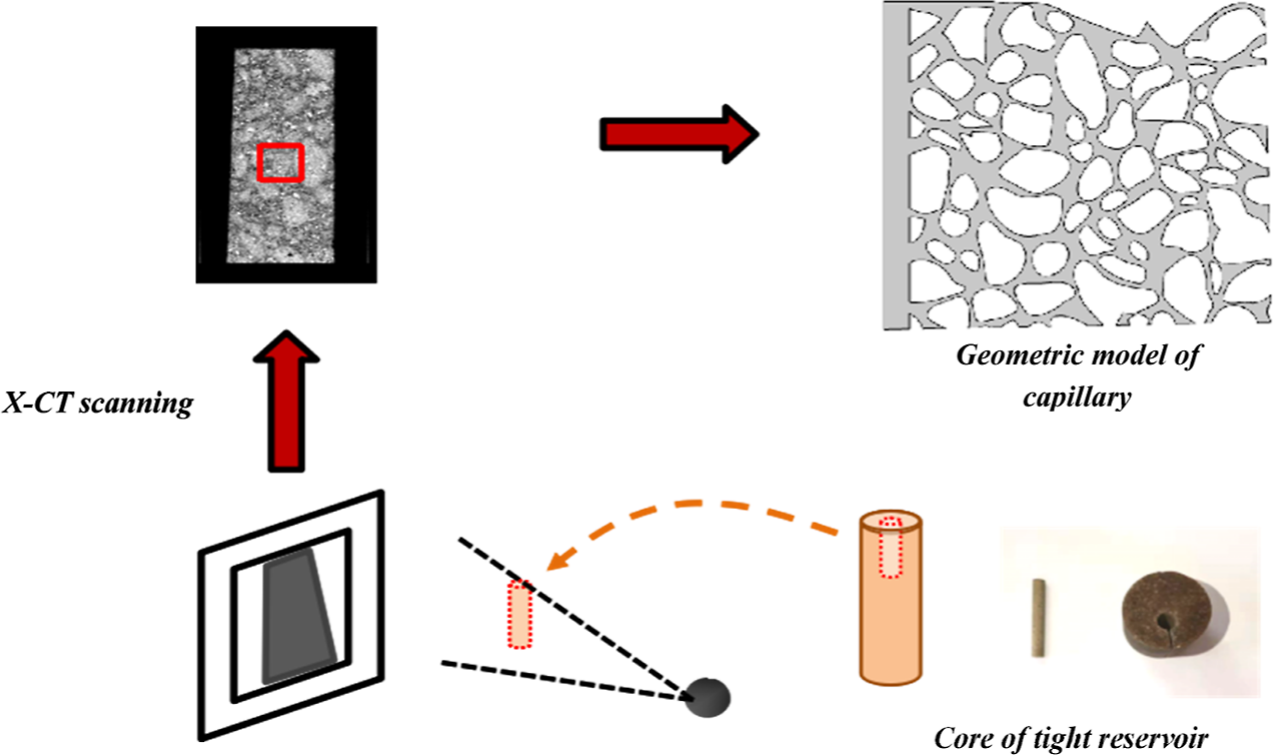
Geometric model of porous media.

Using X-ray computed tomography, a geometric model of the porous media with a model size of 800 μm × 800 μm was created (Figure 2). By combining the Stokes’ equations with the phase field equations, the oil distribution of various surfactants was estimated.

Fluid flow in porous media satisfies the Stokes’ conservation of the momentum equation, ignoring inertial forces (eq 1).
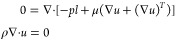
1where *p* is the density(kg/m^3^); μ is the dynamic viscosity (N·s/m^2^); *u* is the velocity (m/s); and *p* is the pressure (Pa).

The oil–water phase field distribution considering the interfacial tension satisfies eq 2.
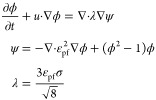
2where ϕ is the phase field variable with an oil phase value of 1 and a water phase value of −1, ε_pf_ is the interfacial thickness, and σ is the interfacial tension coefficient, N/m.

The oil–water interfacial force affects the oil–water phase distribution, and the interfacial force *F*_st_ can be expressed by eq 3.
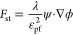
3

Density and viscosity can be expressed by eq 4.
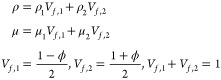
4where *V*_*f*,1_ and *V*_*f*,2_ are the volume fractions of oil and water, respectively.

The initial conditions are as follows
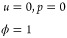
5

The wall boundary conditions are
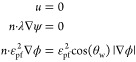
6where θ_w_ is the wetting contact angle and *n* is the normal vector.

The entrance conditions are
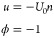
7where *U*_0_ is the inlet flow rate, m/s.

The export conditions are
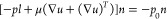
8where *p*_0_ is the outlet pressure, Pa.

### 2.2 Surfactant Huff-n-Puff Production Solution Methodology

The CMG-STARS simulator is a finite difference numerical simulator used to solve a set of conservation equations such as material balance equations, flow equations, chemical reactions, heat-exchange equations, and phase equilibrium equations. The STARS simulator provides accurate calculations of the flow characteristics of surfactants in tight reservoirs.^28^ The simulator utilizes relative permeability curve interpolation to explain the rock fluid flow characteristics in the presence of the surfactant. Interpolation of the capillary number function is required when interfacial tension is a critical component. In the simulator, the capillary number is determined using Darcy’s law rather than velocity. Therefore, the viscosity is offset and the capillary number is calculated as
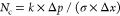
9

The interpolation formulas for the capillary number with known relative permeability values of groups A and B are as follows
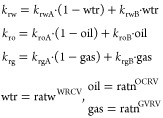
10where ratw and ratn are the values of dimensionless interpolation parameters, which vary between 0 and 1. WRCV, OCRV, and GVRV are the curvature interpolation parameters. The interpolation parameter values are related to the number of capillary tubes.
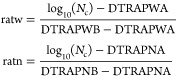
11where DTRAPW and DTRAPN are the interpolation parameters for the low capillary number with high IFT and the high capillary number with ultralow IFT, respectively.

## 3 Experimental Section

### 3.1 Materials and Reagents

#### 3.1.1 Oil and Surfactant

The oil used in this experiment was provided by the T21 block of the no. 9 Oil Production Plant of Daqing Oilfield. The density and viscosity of crude oil were measured by model DMA 4200 M, Anton Paar, Austria and model DV-II+, Brookfield, USA, respectively. At standard atmospheric pressure, the density is 856.3 kg/m^3^ and the viscosity is 2.21 mPa·s at 60 °C.

Surfactants were prepared with 0.5% nonylphenol polyoxyethylene ether (NPE), 0.25% linear alkyl benzene sulfonic acid (LABSA), and 0.25% sodium alkyl ethoxysulfate mixture (AES) at the experimental concentration. Surfactants were prepared with experimental concentrations of 0.5% NPE, 0.25% LABSA + 0.25% sodium ethoxylates (AES), and 0.25% LABSA + 0.25% coconut oil fatty acid (CA). These surfactants were produced by Qingdao USOLF Company. The experiment was carried out using the 0.5% DGN-1 surfactant, which is commonly used in the field.

#### 3.1.2 Cores

In the experiment, core samples from tight oil reservoirs with similar permeability and porosity were utilized. The effect of diverse core samples on surfactant displacement could be overlooked. Two different diameters of cores were applied to measure the relative permeability curve of the surfactant (SRPC) and experiment with surfactant huff-n-puff production (SHP). Table 1 displays the core’s features.

**Table 1:** Basic Core Properties for Experiments

core	experiment type	displacement surfactant	core size	permeability, ×10^–3^ μm^2^	porosity, %
#1	SRPC	NPE	D 2.5 cm × 9.68 cm	1.42	14.24
#2		LABSA + AES	D 2.5 cm × 9.67 cm	1.31	14.92
#3		LABSA + CA	D 2.5 cm × 9.73 cm	1.36	15.30
#4		DGN-1	D 2.5 cm × 9.71 cm	1.33	14.46
#5	SHP	NPE	4.5 cm × 4.5 cm×29.8 cm	1.23	14.62
#6		LABSA + AES	4.5 cm × 4.5 cm×29.3 cm	1.37	14.87
#7		LABSA + CA	4.5 cm × 4.5 cm×29.5 cm	1.42	13.45
#8		DGN-1	4.5 cm × 4.5 cm×29.7 cm	1.28	14.26

### 3.2 Surfactant Huff-and-Puff Productivity Experiment

The water permeability was measured at 60 °C using a steady flow rate, and the saturated oil was aged for 24 h. The gripper was used to conduct surfactant flooding at a steady flow rate of 0.1 mL/min. The test was halted when the surfactant solution reached the outflow end of the core, and the amount of surfactant injected was recorded. The direction of fluid flow was the path of surfactant displacement oil. Equation 12 was used to calculate the percolation rate of various surfactants.
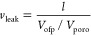
12where *v*_leak_ is the percolation rate of the surfactant, m/PV, *V*_ofp_ is the injection volume at the stage of no water production, mL, *V*_poro_ is the core pore volume, mL, and *l* is the core length, m.

**Figure 3:**
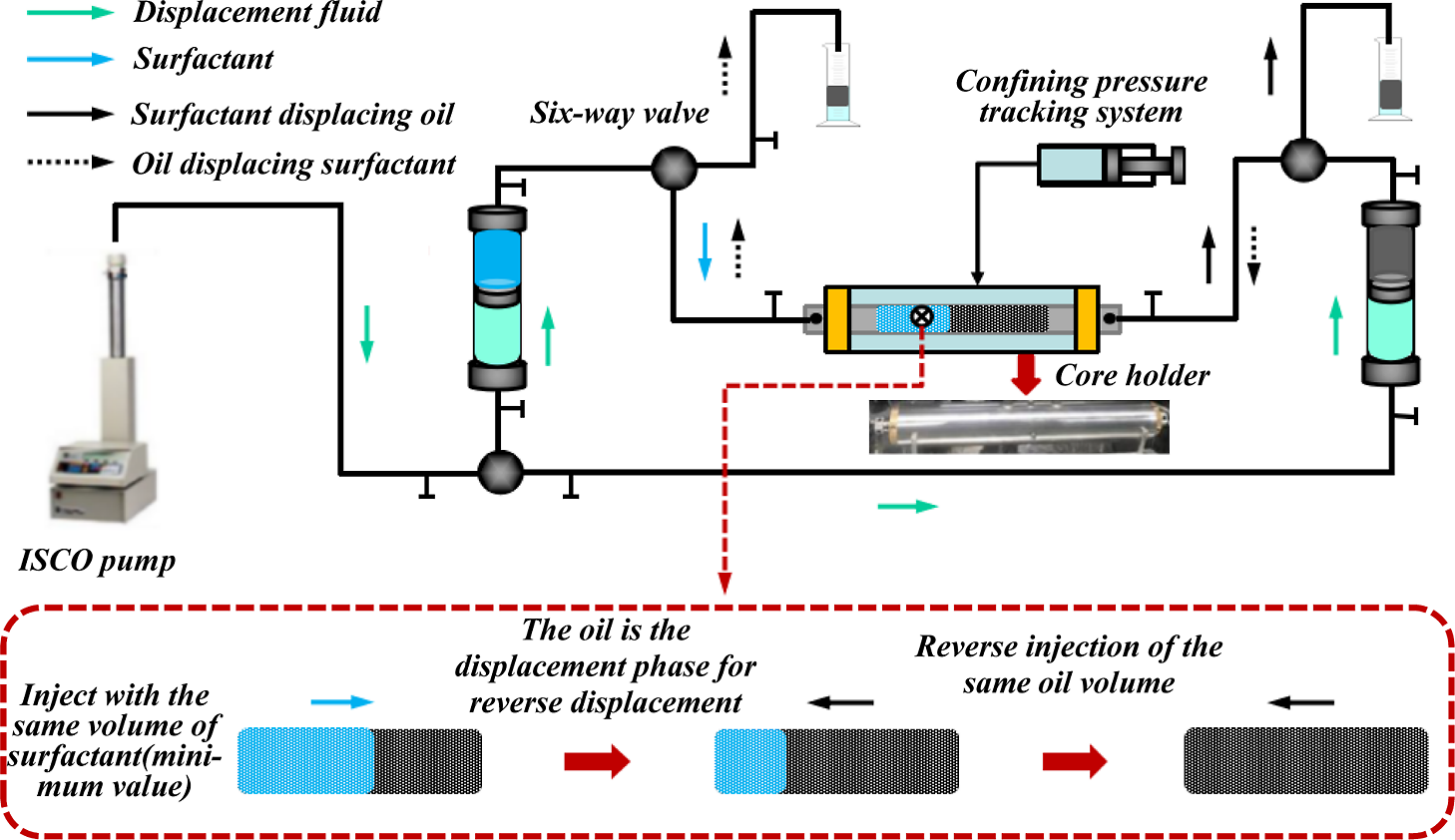
Experimental flow of surfactant huff-and-puff productivity.

The core was cleaned and resaturated with oil. Various surfactants were injected into the core at the minimum volume. To simulate the well-production process, the injection direction was changed and oil was injected into the core. The experimental flow is shown in Figure 3.

### 3.3 Surfactant Relative Permeability Curve

The Johnson, Bossier, and Naumann unsteady method was used to measure the surfactant’s relative permeability curve and determine the surfactant’s displacement efficiency. Constant velocity injection was used to set the pressure threshold at the core inlet. The volume of oil, liquid produced, and pressure difference were recorded in the experiment. Equation 13 is used to calculate the relative permeability of oil and water.
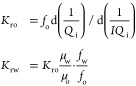
13where *Q*_i_ = *V*/*V*_p_, , *f*_w_ = 1 – *f*_o_, , and *I* = Δ*p*_0_/Δ*p*.

## 4 Results and Discussion

### 4.1 Surfactant Microscopic Flow Characteristics

**Figure 4:**
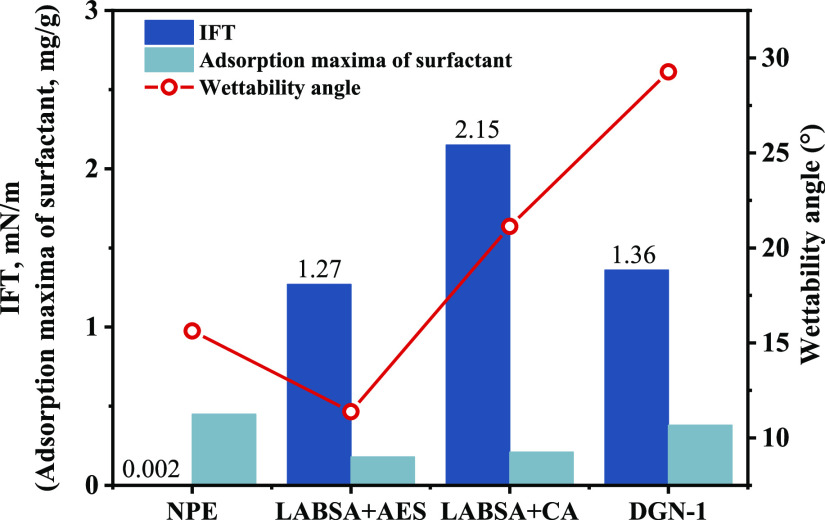
Rock-fluid properties of the surfactant.

The interfacial characteristics of several surfactants were measured (Figure 4). Various surfactant groups have different adsorption effects on the rock surface and solubilization of the crude oil for the same crude oil. As a result, surfactants with various interfacial characteristics were produced.^29^ The distribution states of the fluids within the porous medium during the injection process for each type of surfactant in 3 s were calculated according to Section 2.1.

**Figure 5:**
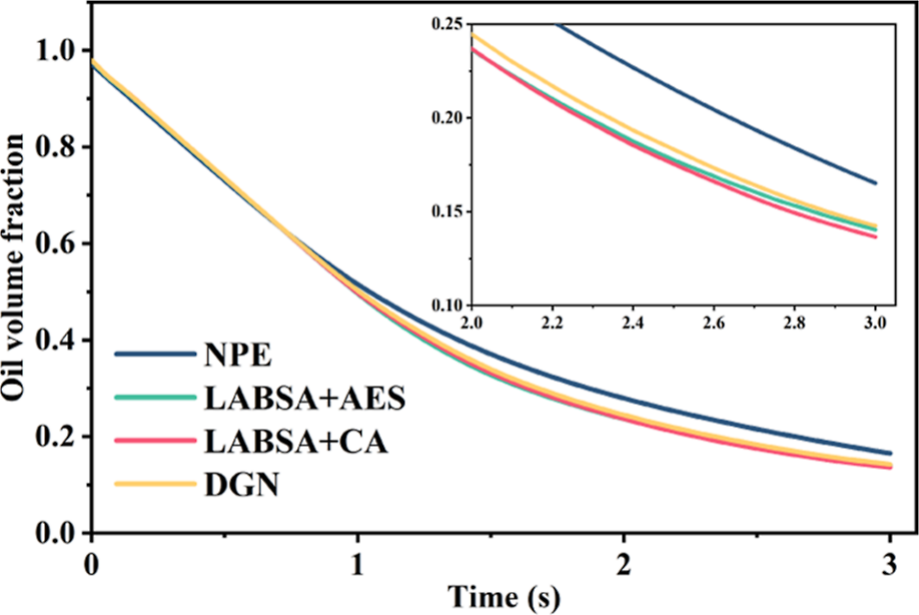
Variation of the volume fraction of different surfactants.

**Figure 6:**
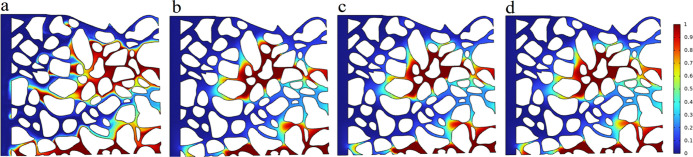
Oil volume fraction distribution of each surfactant after 3 s injection, where, (a–d) are NPE, LABSA + AES, LABSA + CA, and DGN-1, respectively.

**Figure 7:**
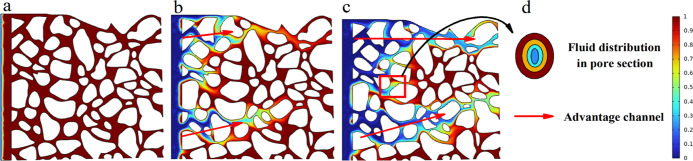
Oil volume fraction distribution of NPE at different times. (a–c) Oil volume fraction distributions at 0, 1, and 2 s, respectively and (d) fluid distribution in the pore section.

The interfacial properties of surfactants affect the oil distribution state. The largest volume fraction of oil is retained after NPE injection, while LABSA + CA corresponds to the minimal remaining oil (Figure 5). The differences in interfacial tension (Figure 6a,d) compared to the variation in wetting angle (Figure 6b,d) had a greater effect on the oil remaining in the pore space. Figure 7 demonstrates that the NPE surfactant has ultra-low interfacial tension (<10^–2^ mN/m) with crude oil and a significant wetting angle (12.6°) with rock surface, which makes it flow via large-sized pores and establishes dominating pore channels in the formation. This enables more crude oil to remain trapped in the fine pores. Its percolation rate is faster. The NPE flows through into the centre of the pore as the oil is adsorbed by the pore wall, resulting in a progressive rise in the volume fraction of oil in the pore cross-section from the inside to the outside (Figure 7d).

### 4.2 Model Validation through History Matching

**Figure 8:**
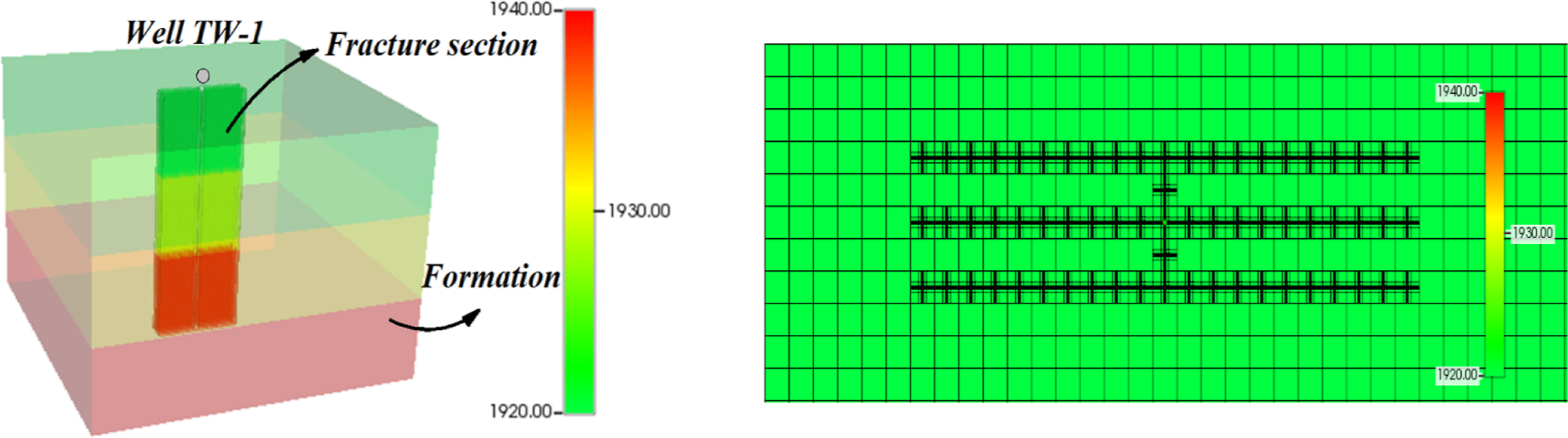
Three-dimensional model of the surfactant fracturing model.

The 3-D model describing the surfactant huff-puff process has been shown in Figure 8. The fluid was injected from the bottom of the produced well and diffused along the fracture. The model was divided into 50 × 50 × 3 grids, with each grid in the *i*, *j*, and *k* directions having dimensions of 20, 20, and 10 m, respectively. The model size corresponds to the location of the T21 block where the TW-1 well was actually produced. The actual production well was exploited for 600 days after initial large-scale fracturing with surfactant DGN-1. The fracture grid scale of well TW-1 was detected by ground microseismic detection. The detection results are shown in Table 2. The DGN-1 surfactant properties (Figure 4), relative permeability data (Figure 10), and basic parameters of the model parameters (Table 3) were assigned. The calculation results were in comparison to well history to verify the model.

**Figure 9:**
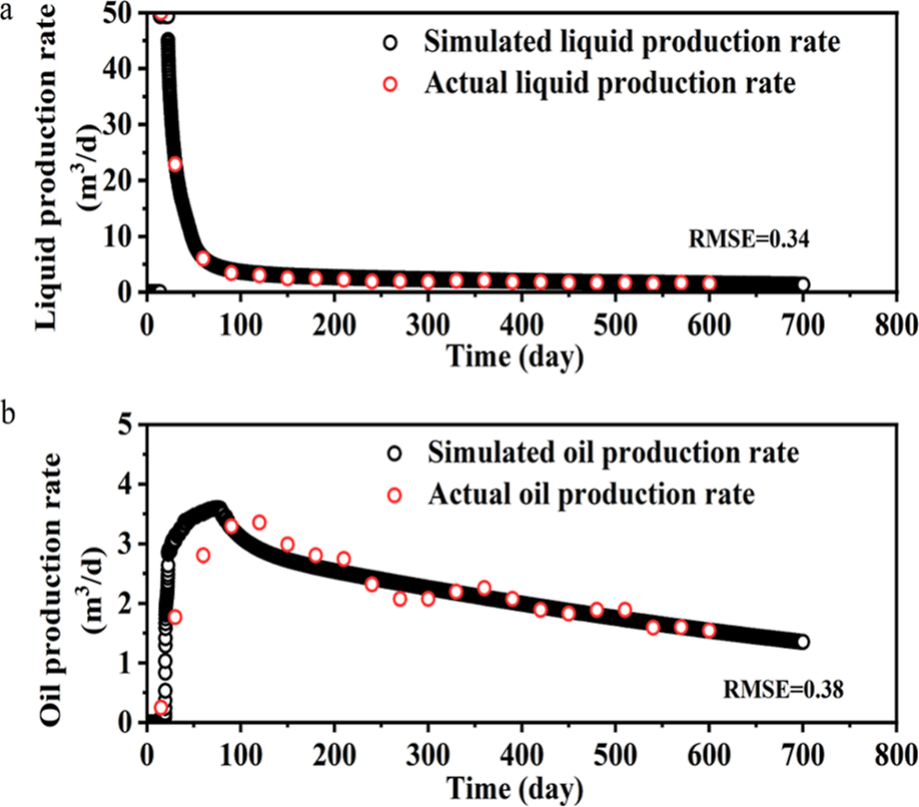
Production curve based on simulation and actual production. (a,b) Fitted curves of daily liquid production and daily oil production, respectively.

**Figure 10:**
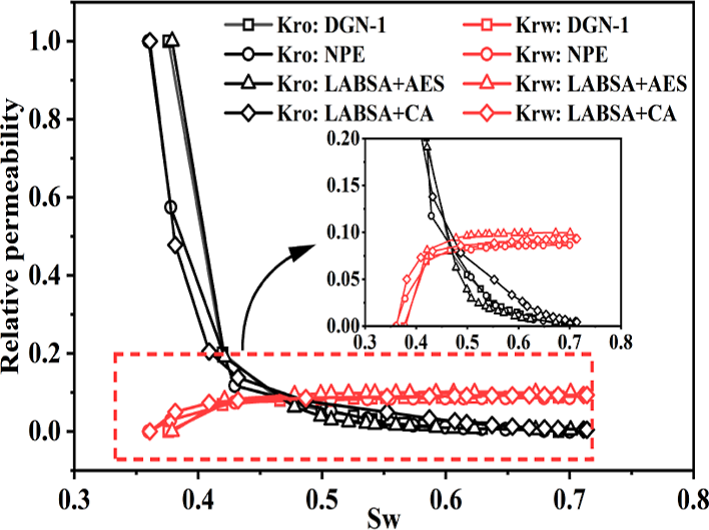
Relative permeability curves of surfactants.

**Table 2:** Ground Microseismic Detection Results

detection results	value
perforation zone	2074.2–2077.6 m
fracture zone width	96 m
fracture length	486 m
fracture height	36 m
fracture azimuth angle	NW80°

**Table 3:** Basic Parameters of the Model

reservoir model parameters	value
permeability	1 × 10^–3^ μm^2^
porosity	13%
initial oil saturation	0.64
depth	1920 m
pressure	20.36 MPa
temperature	60 °C
thickness	30 m
fracture zone width	100 m
fracture length	400 m
injection strength	7.91 m^3^/min
bottom hole pressure	8.2 MPa

It can be seen from Figure 9 that the simulation results are highly consistent with the actual results. Liquid production decreases rapidly. The simulation results represent the actual situation after adjustment. This model can be used for subsequent research.

### 4.3 Rock-Surfactant Percolation Characteristics

**Figure 11:**
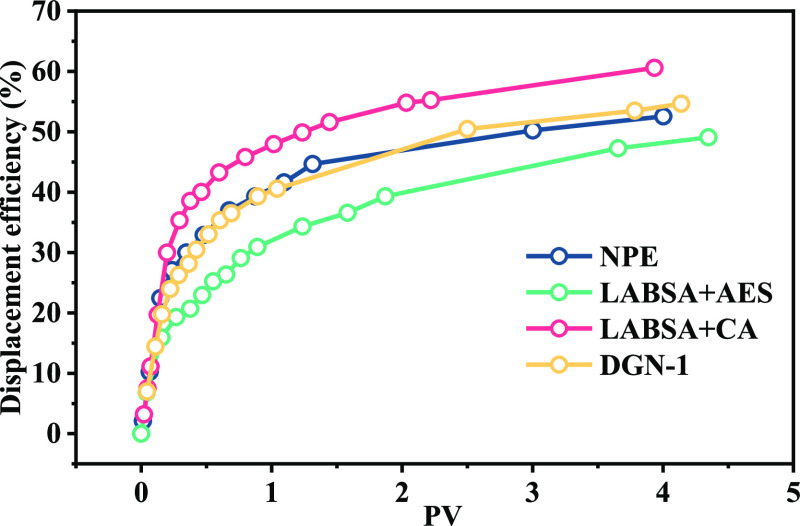
Displacement efficiencies of surfactants.

The surfactant–oil relative permeability curve was calculated using the approach given in Section 3.3. The application of surfactants resulted in the water saturation being in a range of 0.36–0.72 in the two-phase flow region. Displacement efficiency was also obtained (Figure 11). The displacement efficiencies of various surfactants range from 49 to 61%. Surfactant displacement effectiveness correlates with oil relative permeability. LABSA + CA exhibited the highest displacement effectiveness and the slowest surfactant percolation rate among them.

### 4.4 Percolation Rate and Production Characteristics of Surfactant Huff-n-Puff

The surfactant percolation rate and production were evaluated by the experiment on surfactant huff-n-puff, which was compared with the results in the STARS simulator. Table 4 reveals that the longer the no water production period, the slower the percolation rate. NPE has the fastest value, which reaches 2.8 m/PV. The relationship between percolation rate and oil production is not linear. The percolation rate has a significant impact on oil production, and there is a range of optimum values.

**Table 4:** Result of the Experiment on Surfactant Huff-n-Puff

displacement surfactant	injection surfactant volume at the stage of no water production, mL	percolation rate, m/PV	oil production, mL
NPE	6.42	2.58	1.50
LABSA + AES	10.25	1.63	3.80
LABSA + CA	16.44	1.02	0.58
DGN-1	13.16	1.38	1.33

It is necessary to demonstrate the production of surfactant huff-n-puff by the water cut because of the different sizes of experiment and numerical model construction.

**Figure 12:**
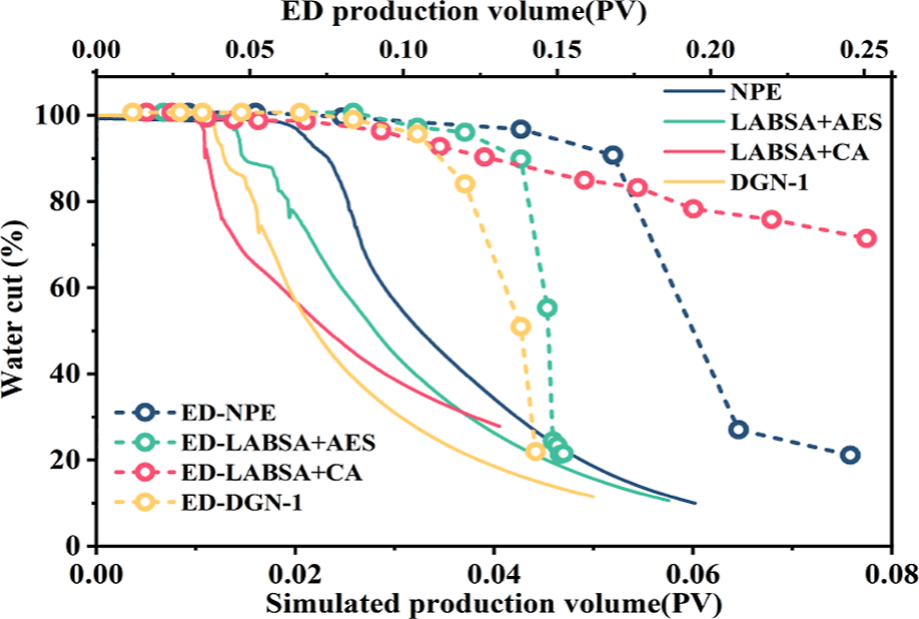
Numerical model and experimental water cut curve.

**Figure 13:**
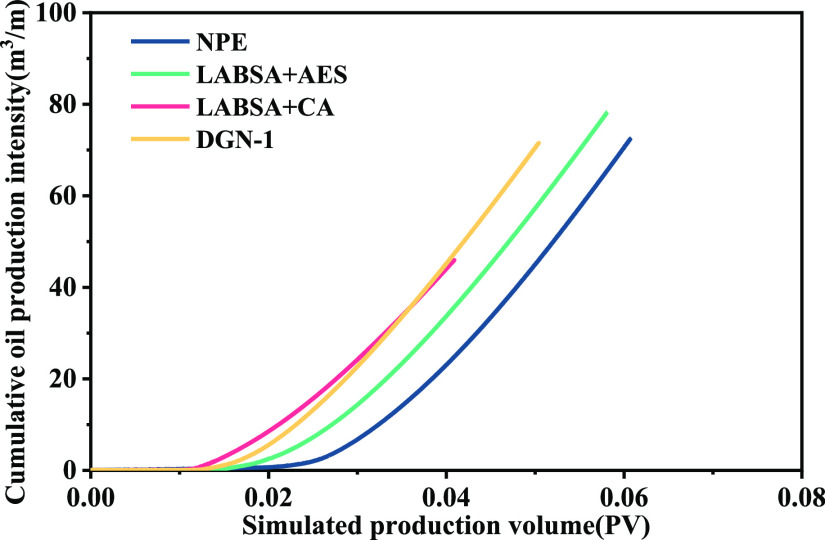
Simulation calculation of cumulative oil production.

Figure 12 depicts the strong consistency of the productivity variation between the simulation calculation and the laboratory experiment. LABSA + CA initially terminates the stage of oil-free water production. Its water cut is dramatically reduced at first because the surfactant has the slowest percolation rate and the highest oil displacement efficiency. This results in the shortest percolation distance, and the oil around the crack flows into the well first during production. The oil pushed away during huff-n-puff is trapped in the pores, and the water cut decreases slowly. On the other hand, surfactant NPE has the quickest seepage velocity and the percolation range is far from the well. Despite significant liquid production, some oil is pushed further out, resulting in the oil being unable to migrate properly in the drainage region (Figure 13). The interfacial characteristics of LABSA + AES and DGN-1 were comparable, and their percolation patterns were similar. High huff-n-puff production for their percolation rates (1.38–1.63 m/PV) was attained.

### 4.5 Productivity Variation Characteristics under Different Injection Parameters

In order to obtain the influence of injection volume and injection intensity (rate of surfactant injection under unit thickness of the reservoir) on the productivity of huff-n-puff, the variation characteristics of oil production intensity and cumulative oil production intensity were calculated, respectively.

**Figure 14:**
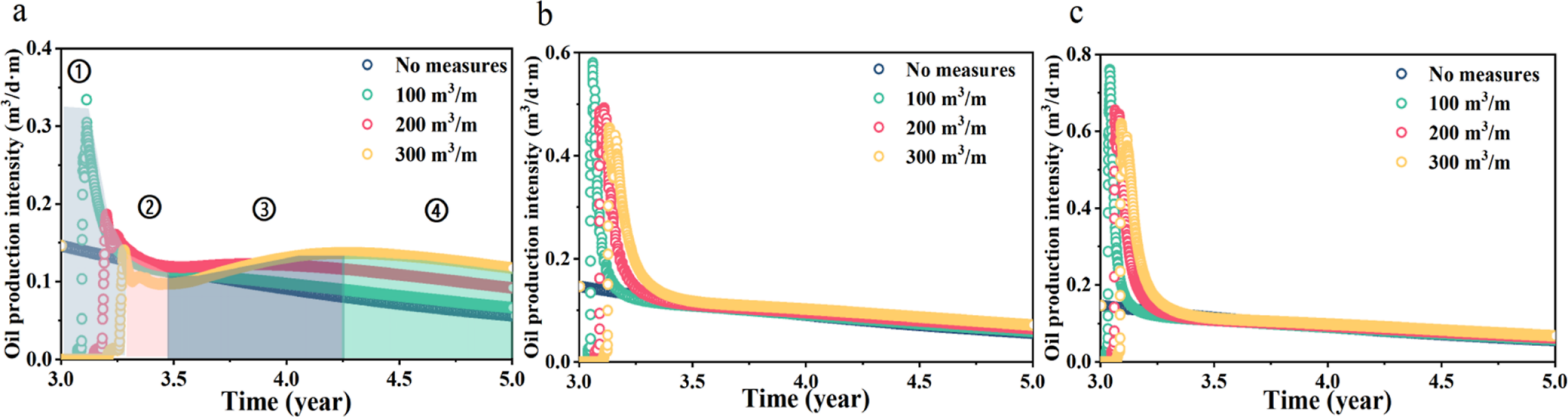
Daily oil intensity under different injection parameters. (a–c) Daily oil intensity of 4 m^3^/(m·d), 8 m^3^/(m·d), and 12 m^3^/(m·d), respectively.

The huff-n-puff was implemented after the first fracturing production for 3 years. The variation characteristics of daily oil production intensity in Figure 14a can be divided into four stages. An injection volume of 300 m^3^/m was given as an example. The first stage is the surfactant return stage, also described as the oil-free water production stage. With the return of the surfactant, oil production increases and starts to decline rapidly after reaching its peak. This process is the decay production stage after energy enhancement. The surfactant entering the formation through the fracture provides the primary production energy for this process. The injection intensity is proportional to the maximum daily oil intensity. The surfactant production enhancement stage occurs when the surfactant in the far-well area flows to the near-well area after energy is released through percolation and pressure. This causes an increase in oil production. Combining Figure 14b,c, it can be seen that this stage increases the seepage range due to high injection volume and low injection intensity, which makes the increase in oil production more obvious. Then, it enters the natural decreasing stage, and the oil recovery intensity gradually decreases.

**Figure 15:**
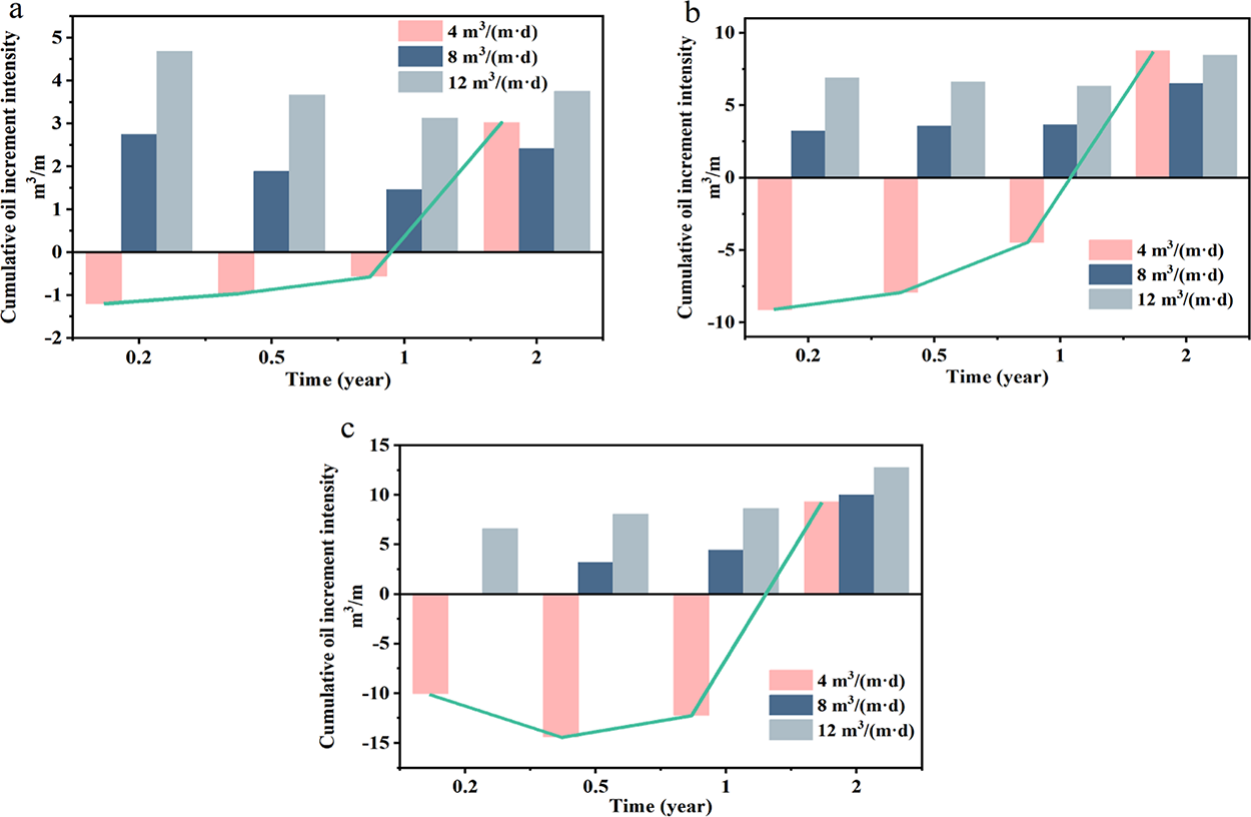
Curve of cumulative oil increment intensity versus time. The injection volumes of (a–c) are 100, 200, and 300 m^3^/m, respectively.

The cumulative oil increment intensity was calculated, which is the difference between the current cumulative oil production with and without surfactant injection per unit reservoir thickness. The injection intensity has a significant impact on the oil increment effect. At the same injection volume, the rise amplitude at the initial stage of production is positively correlated with the injection intensity; however, the difference in value becomes smaller as the stage progresses. Figure 15a shows that injecting 4 m^3^/(m·d) increases the seepage distance, but the production capacity within a year is lower than it would be in the absence of measures. The period of the low production stage increases as the injection volume increases (Figure 15b,c). The oil increment of 8 m^3^/(m·d) injection intensity at a volume of 200–300 m^3^/(m·d) is increasing, and 12 m^3^/(m·d) injection intensity produces different effects depending on the volume. To explain this phenomenon, two notions are proposed: the limit injection volume and the optimal injection volume. The limiting injection volume and the optimal injection volume are the parameters corresponding to the time dependence when the cumulative oil increase intensity is 0 and the maximum value, respectively. These two concepts are used to describe the relationship between surfactant huff-puff injection intensity, injection volume, and oil production.

**Figure 16:**
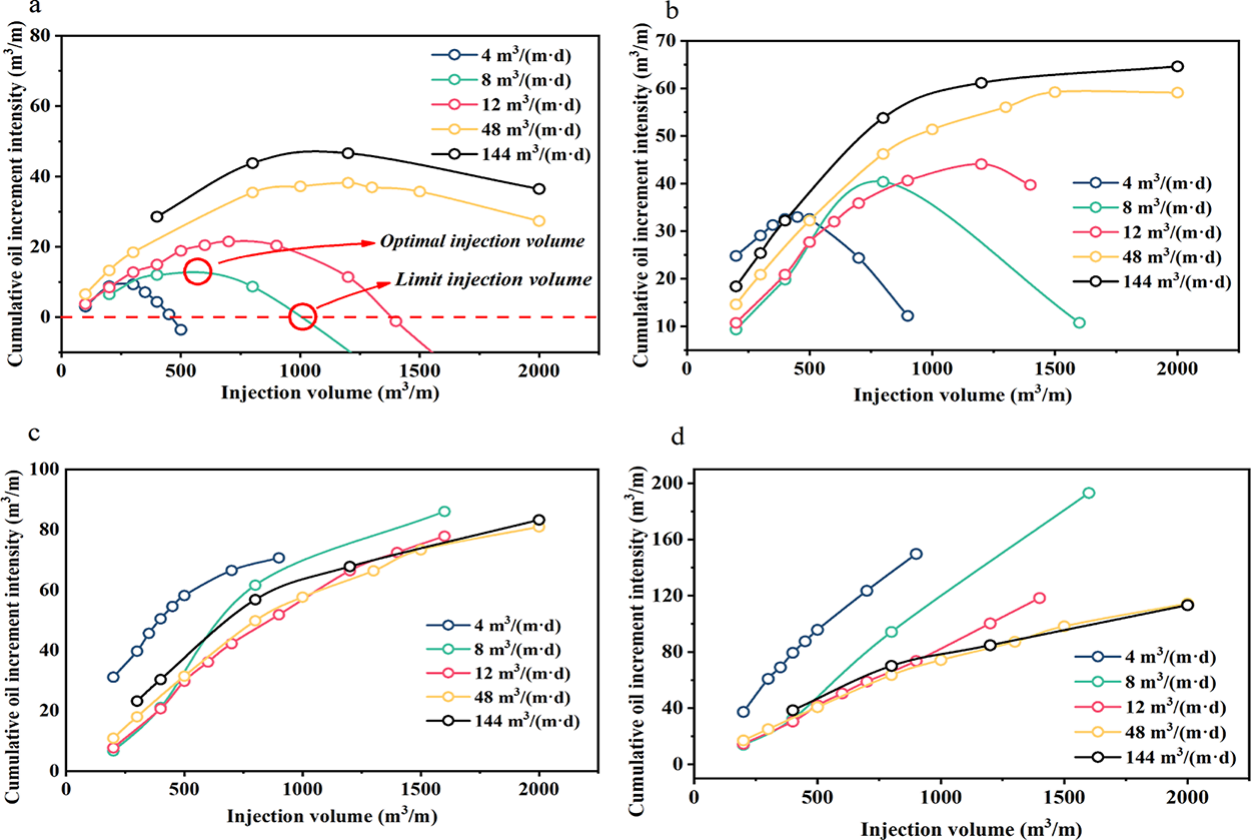
Cumulative oil increment intensity per unit reservoir thickness under different injection rates, where (a–d) are curves for 2, 3, 5, and 7 years, respectively. Note: combined with field fracturing measures, in surfactant huff and puff, the injection strength is less than 144 m^3^/(m·d), and the fracture is not expanded.

Figure 16 illustrates that the optimal injection intensity in a short production time is less than the limit injection volume. It is difficult to obtain the limit injection volume after 5 years. Similarly, as injection intensity decreases, it becomes easier to attain the limit and optimal injection volume. From the perspective of production capacity, it is necessary to improve the injection intensity in order to obtain a high oil increase in a short time, but increasing the injection intensity leads to the increase in the optimal injection volume, which is basically maintained in the range of 1000–1200 m^3^/m. The increase in oil production is restricted when the injection intensity is more than 48 m^3^/(m·d). Figure 16-d shows that when the injection volume is 1000 m^3^/m, the injection intensity of 12–144 m^3^/(m·d) has no change after 7 years. However, with effective fractures and long-term production, a higher recovery can be reached with lesser injection strength.

**Figure 17:**
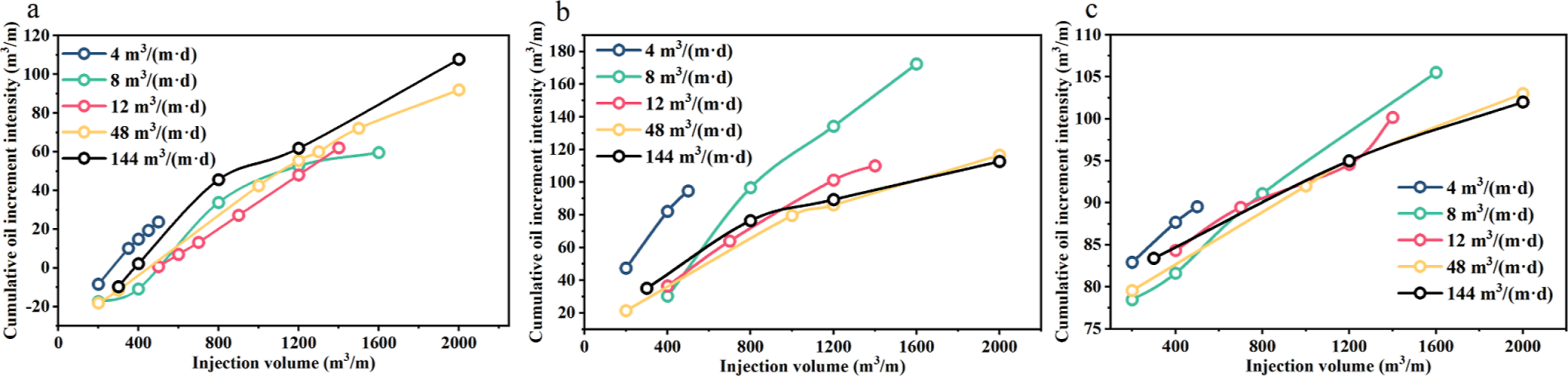
Oil increase in different surfactants, where (a–c) are the cumulative oil increment intensity under different injection of NPE, LABSA + AES, and LABSA + CA in 5 years, respectively.

As shown in Figure 17, the production variation of LABSA + AES and LABSA + CA is consistent with DGN-1, where LABSA + AES has the highest huff-n-puff production. To achieve high production, NPE requires a higher injection intensity. When combined with the percolation rate in Table 4, NPE needs to increase the injection intensity to reduce the seepage area and increase the formation energy around the fracture for production. Lower percolation rates of LABSA + CA, on the other hand, have the highest displacement efficiency, resulting in lower oil saturation around the fracture and oil-rich zones further away from the well. Therefore, when selecting surfactants for huff-puff production, their displacement efficiency and percolation rate need to be considered.

## 5 Conclusions

In general, the implementation of secondary surfactant huff-n-puff can extend the effective production time. Ignoring the variation of fracture morphology and effective time, surfactant percolation rate and displacement efficiency have a significant impact on huff-n-puff production. Surfactants with high displacement efficiency are not conducive to production. There are limit and optimal injection volumes in the surfactant injection procedure. They pointed out that in order to obtain a higher production in a short time, higher injection intensities are required and correspond to optimal injection volumes. The present research can enrich the study of EOR in tight reservoirs.

## Nomenclature

*Q*_i_: cumulative injection, mL
*I*: flow capacity ratio, dimensionless
*K*_ro_: oil-phase relative permeability
*K*_rw_: water-phase relative permeability
*f*_o_: oil-phase split rate, fraction
*f*_w_: water phase split rate, fraction
μ_o_: oil-phase viscosity, mPa·s
μ_w_: water-phase viscosity, mPa·s
*V*: cumulative injection, mL
*V*_p_: core pore volume, mL
average water saturation, fraction
*S*_wi_: initial water saturation, fraction
*V*_o_: cumulative recovery
Δ*p*_0_: initial pressure difference (difference in measuring oil phase permeability under bound water as the initial pressure difference in water flooding), MPa
Δ*p*: pressure difference, MPa
